# Reinstatement of memory representations for lifelike events over the course of a week

**DOI:** 10.1038/s41598-017-13938-4

**Published:** 2017-10-30

**Authors:** Christiane S. H. Oedekoven, James L. Keidel, Sam C. Berens, Chris M. Bird

**Affiliations:** 1 School of Psychology, University of Sussex, Brighton, UK; 2 Department of Psychology, University of York, York, UK

## Abstract

When we remember an event, the content of that memory is represented across the brain. Detailed memory retrieval is thought to involve the reinstatement of those representations. Functional MRI combined with representational similarity analyses (RSA) of spatial patterns of brain activity has revealed reinstatement of recently-experienced events throughout a core memory retrieval network. In the present study, participants were scanned while they watched, immediately retrieved and then retrieved after a week, 24 short videos. Following the delayed retrieval, they freely recalled all videos outside of the scanner. We observed widespread within- and between-subject reinstatement effects within a posterior midline core memory retrieval network during all phases of the experiment. Within precuneus, bilateral middle temporal gyrus and the left hippocampus, reinstatement effects between the retrieval phases correlated with memory performance. These findings extend previous studies that have only employed short retention periods or highly rehearsed materials, demonstrating that memory representations for unique events are reliably reinstated over longer timeframes that are meaningful in the context of real-world episodic memory.

## Introduction

Episodic memory is the recollection of lifelike events^1^. Very recent events can often be recollected in great detail, but specific details may be quickly forgotten, leaving only a vague and gist-like memory^1–4^. Nevertheless, some memories are retained in detail for weeks and even years^5,6^. When we retrieve an episodic memory, it is thought that we reinstate neural representations that were present when the event was encoded^7–9^. In addition to enabling detailed and vivid recall of the event, this active retrieval may promote consolidation of the event^10^. Although reinstatement effects for complex lifelike stimuli such as video clips have been observed over short retention periods^11–13^ or over long delays using highly practiced stimuli^14^, reinstatement of individual episodic memories has not been demonstrated over the timeframes in which episodic memory operates in everyday life. In this study we investigate memory reinstatement for detailed lifelike memories between encoding, immediate retrieval and one-week delayed retrieval.

Most neuroscientific models of systems memory consolidation argue that memory traces are stabilized by neuronal reactivation of memory traces, whereby the reinstatement of patterns of neural firing elicited during encoding strengthens links between the hippocampus and distinct cortical areas or between cortical regions themselves^15–18^. Although this is often viewed as a passive process, some have argued that active retrieval may promote faster consolidation of memories^10^. Several functional MRI studies have found evidence for this reactivation by showing that spatial patterns of fMRI BOLD activity are similar when people encode and retrieve the same memories. This is the case for simple stimuli such as picture-picture or word-picture pairings^19–24^ as well as more complex stimuli such as short videos^11,13,14^ or events from a continuous film^12^. Reinstatement effects between encoding and retrieving episodic memories^11–13^ are usually seen within a “core retrieval network” including the hippocampus/parahippocampal gyrus, the angular gyrus (AG) and posterior midline cortex (PMC) including precuneus and posterior cingulate cortex (PCC^25,26^), a group of regions which has also been characterized as the Posterior Medial system (PM System^27,28^; see also^29^ for a similar chracterization). Bird *et al*.^11^demonstrated that the degree of reinstatement in the PCC is related to subsequent memory performance, suggesting a particularly important role for this structure in successful memory retrieval.

Beyond these within-subject findings, a recent study also reported inter-subject correlations in the core retrieval network across subjects watching and retrieving aloud a 50-minute film^12^, suggesting the existence of “shared” representations of the contents of specific scenes. Within PMC and other parts of the core retrieval network, these inter-subject pattern correlations were higher during retrieval than between encoding and retrieval, suggesting that memories had been altered in a systematic way across participants to representations that were more generic and schematic in nature.

Reinstatement effects have typically been investigated across retention intervals of less than a day. Investigating episodic memories over longer retention intervals presents a challenge as they are typically forgotten relatively quickly^2,3^, and even lifelike stimuli such as videos are remembered relatively poorly after a week if they are not rehearsed^11,30^. To circumvent this issue, Buchsbaum and colleagues^14^ scanned a group of participants who were highly practiced at mentally replaying a large number of 5-second videos and showed that patterns of BOLD activity during mental replay resemble those present during encoding (see also^31^). Using longer videos, Bird *et al*.^11^ showed that a single retrieval phase soon after encoding resulted in memories that could be recalled in detail one or two weeks later. Very similar findings were reported by Sekeres *et al*.^30^. This benefit of an immediate retrieval phase on subsequent recall performance has been reported for a wide variety of memory stimuli and is referred to as the “testing effect” or “retrieval practice effect”^32–34^. The design of our study allows us to investigate this phenomenon in greater depth, as participants were scanned during memory encoding, immediate retrieval (retrieval practice) and delayed retrieval after a week. Our study had several aims. The first was to replicate previous studies demonstrating reinstatement between encoding and immediate retrieval. The second was to investigate reinstatement between encoding and delayed retrieval, extending the findings of Buchsbaum and colleagues^14^. The final and most important aim was to investigate reinstatement between immediate and delayed retrieval and to compare this to reinstatement effects between encoding and retrieval.

In general, we predict that retrieval of lifelike events involves the reinstatement of spatial patterns of BOLD activity that were present during encoding, irrespective of the retention delay. However, it is possible that reinstatement between the retrieval phases will be greater than between encoding and retrieval. Retrieval practice is argued to result in the establishment of elaborated or transformed memory traces rather than simply strengthening or stabilizing the original traces^33,35–37^. In this case (a) there should be additional regions that exhibit reinstatement between retrieval phases compared with encoding and retrieval phases, or (b) there should be significantly greater reinstatement between retrieval phases in at least a subset of the regions where there is reinstatement between encoding and retrieval phases.

Following previous studies, we use representational similarity analyses (RSA^38^) to investigate reinstatement effects when encoding and retrieving matching videos as well as to identify regions where reinstatement correlates with memory performance a week after encoding. Whole brain “searchlight” analyses are reported which show the entire distribution of the effects, as well as effects within a PMC region of interest (ROI) given this area’s consistent implication in memory reinstatement effects. Our main focus will be on reinstatement effects between the two retreival phases, including where reinstatement correlates with subsequent memory performance. Additionally, we report findings from inter-subject pattern correlation analyses that replicate and extend the recent findings of Chen and colleagues^12^.

## Methods

### Participants

Twenty-five adults participated in the study but four were excluded from further analyses: one due to excessive head motion, one due to failure to remember the videos and two due to technical problems leading to incomplete data sets. We report results from 21 young adults (aged 18–35, mean age 25.6 SD 5.1 years, 11 female). All were healthy adults, right-handed and had normal or corrected to normal vision. They gave written informed consent and were paid for participation. The study was approved by the Brighton and Sussex Medical School Research and Governance Ethics Committee and was conducted in accordance with relevant guidelines and regulations.

### Experimental design

Participants watched 24 short videos while in the MRI scanner. The videos lasted on average 38 seconds (range 29–48 s) and were taken from short films or videos posted on www.YouTube.com. All videos depicted a short narrative and were presented without sound. The stories centered around one character (5 videos), two main characters (8 videos) or an interaction of multiple characters (11 videos). 13 videos took place outside, 8 videos took place inside of a building and three videos switched between these types of locations. The order of the videos was pseudorandomized across participants. All stimuli were pre-experimentally unfamiliar to the participants.

**Figure 1:**
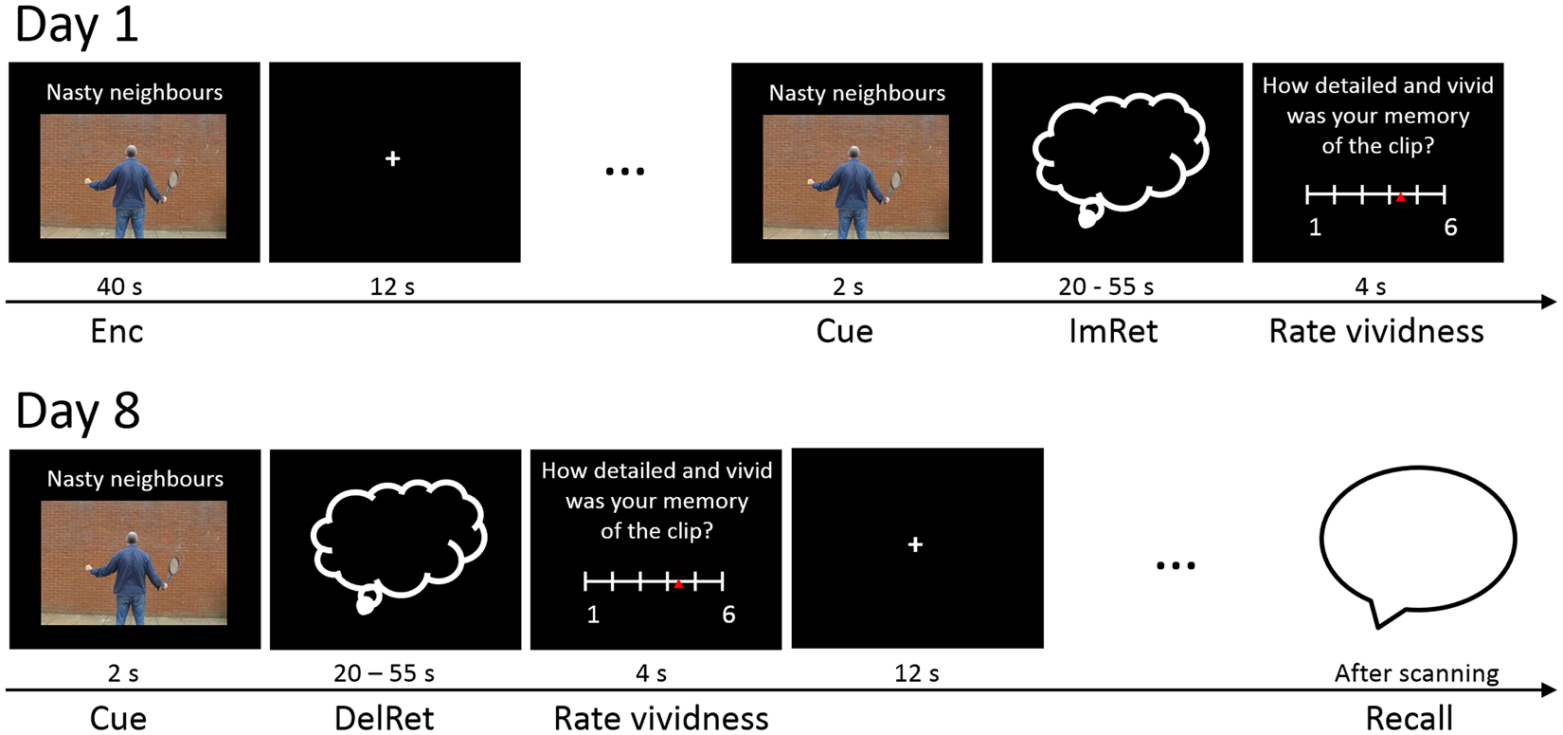
Study design. On Day 1 there were two scanning runs. In each run, participants consecutively watched all 12 videos (encoding phase, Enc), and then were cued to silently retrieve each video (immediate retrieval phase, ImRet) and then rate the vividness of their memory. Cues were the titles of the video and a screenshot showing the first frame. One week later (Day 8), participants silently retrieved all 24 videos (delayed retrieval phase, DelRet) and rated the vividness of each memory in a single scanning run. After scanning, they described all 24 videos to the experimenter.

All participants were scanned twice, with one week between scans. Participants were scanned at a similar time of day on both days. The task was programmed in the Cogent 2000 toolbox (http://www.vislab.ucl.ac.uk/cogent_2000) using MATLAB (Version 2013b, The MathWorks, Inc., Natick, MA, USA). For a visualization of the study design, see Fig. 1.

**Day 1**: Before scanning, all participants performed a practice trial with an example video outside of the MRI scanner. In the scanner participants watched (encoding phase: Enc) and retrieved (immediate retrieval phase: ImRet) 24 short videos, split into two scanning runs. In each run participants watched all 12 videos first, and then completed the retrieval phase. We chose to split this phase into two separate scanning runs in order to give the participants a break from the scanner noise, as the experimental portion of the scanning on Day 1 lasted around 45 minutes on average (durations differed across subjects due to the self-paced nature of the retrieval task).

During the Enc phase, each video was presented on a black background and had a title displayed above throughout the presentation which was broadly related to the content of the video (e.g. “Nasty neighbours”). Between each video, a white fixation cross was shown on a black background for 12 s.

Before the ImRet phase, participants saw a 2-second cue consisting of the video’s title and a static image of the first frame of the video; the screenshot then disappeared and the title then faded to grey and but was displayed for the duration of the trial on a black screen. The ImRet phase started as soon as the cue faded. It was self-paced within a duration window of more than 20 s and less than 55 s per trial. Participants indicated when they had finished silently retrieving the video by pressing a button with their right index finger. They were then asked to rate the vividness of their memory on a sliding scale of 1 to 6. After the ISI of 12 s, retrieval of the next video began.

After the scan, participants were requested not to rehearse the videos during the following week until their next scan.

**Day 8**: In a single scanning run, participants again silently retrieved all 24 videos (delayed retrieval phase: DelRet). The DelRet phase was structured identically to Day 1, including a cue and a vividness rating for each memory.

After the scan, participants were asked to describe each video in as much detail as possible to the experimenter. They were cued with the title of the video. If they could not remember the video by the title alone, they were given up to three standardized hints per video. All video descriptions were audiotaped.

### Behavioral data analysis

Memory performance during the final free recall phase outside of the scanner was assessed using the same procedure as Bird *et al*.^11^. This provides a single score corresponding to the amount of detail recalled about each clip and is an objective performance measure that can be used in parametric analyses of the imaging data. Each video’s description was scored for the amount of independent details they contained. For each detail recalled, participants were given a score of 0, 0.5 or 1. The score would be 0 if a detail was not mentioned, it would be 0.5 if a detail was partially correct (e.g. “someone”, “picks up something”) and 1 if the detail was fully correct (e.g. “a man”, “picks up bricks”). There was no maximum amount of details to be recalled per video. This procedure is based on the scoring of widely used prose recall tests (e.g. Rivermead Behavioural Memory Test;^39^). To ensure consistency across participants, all video descriptions were rated by one of the authors (C.O.).

In the parametric analyses of the imaging data (see Within-subject RSA below) we wished to account for the fact that some videos were more memorable than others. For example, a score of 10 might be above the mean for one particular video but below the mean for another. Therefore, the mean number of details recalled across all participants for a video was subtracted from the participant’s score for that video, in order to index the relative memory performance on each video.

All behavioral data were analyzed in SPSS 22 (IBM). We compared retrieval duration and vividness ratings across scanning runs on Day 1 and Day 8 with paired *t* tests.

### MRI scanning

All images were acquired on a Siemens 1.5 T Avanto MRI scanner (Siemens, Erlangen, Germany) using a 32-channel head coil. For the acquisition of fMRI data we acquired T2*-weighted gradient echo-planar imaging (EPI) sequences (TR = 2.62 s, TE = 42 ms, FA = 90°, FoV = 256 mm). We acquired 35 oblique 3-mm-thick slices in ascending order; voxel size 3.0 × 3.0 × 3.0 mm, with 0.6 mm inter-slice gap, aligned parallel to the AC–PC plane. The Enc and ImRet phases were scanned in two runs on Day 1 and the DelRet phase was scanned in one run on Day 8. Anatomical images were acquired on Day 8 using a 3D T1-weighted MP-RAGE sequence (TR = 2.73 s, TE = 3.57 ms, FoV = 256 mm, voxel size 1.0 × 1.0 × 1.0 mm).

### Functional MRI data analysis

We conducted several RSA searchlight analyses^38^ to investigate similarities in spatial patterns of BOLD signal associated with memories for lifelike events over time. All images were analyzed using SPM8 (http://www.fil.ion.ucl.ac.uk/spm) and the CoSMoMVPA toolbox^40^ in MATLAB (Version 2013b, The MathWorks, Inc., Natick, MA, USA). To describe our findings we used MNI coordinates and the Anatomy toolbox for SPM8^41^. Statistical images were cluster corrected for FWE at *p* < 0.05, using a height-defining threshold of *p* < 0.001. Thresholds were determined for whole-brain comparisons as well as for a ROI encompassing the hippocampus and the parahippocampal gyrus bilaterally (HC/PHC ROI, defined in the WFU PickAtlas, Functional MRI Laboratory, Wake Forest University School of Medicine) which was selected based on the findings of Bird *et al*.^11^ and the known importance of this region in episodic memory.

In addition, we performed RSAs on the data from all voxels contained within two independently defined posterior midline ROIs. The PMC ROI (center of mass: 1, −53, 28), encompassing precuneus and PCC, was created from a posterior-medial cluster in the dorsal default mode network and was taken from a study of resting state connectivity^42^ and was used in the study by Chen *et al*.^12^. The mask was transferred into native space and we extracted average Fisher-transformed same–video and different–video correlations from this PMC ROI for each participant. In addition we used the PMC ROI to look at the association of reinstatement with memory performance. To attempt to replicate the finding of Bird *et al*.^11^, who found that the degree of reinstatement correlated with memory performance in a region of the PCC, a second ROI encompassing this region (center of mass: −5, −43, 33) was used for an RSA analysis between Enc and ImRet weighted by memory performance.

#### Preprocessing

The first five volumes of each run were discarded to allow for T1 equilibrium. Functional EPIs were realigned, slice-time corrected to the middle slice and co-registered to the participant’s own anatomical image. These images were used for the RSA analysis.

#### Multivariate analysis

At the first level all runs were modeled separately and analyses were carried out in native space. On Day 1, there were 36 regressors of interest in each of the two runs corresponding to the 12 Enc trials, the 12 ImRet trials and the 12 screenshot cues at the start of each retrieval. The Enc and ImRet regressors varied in duration according to the length of the video or the self-paced retrieval phase, whereas the cue regressor was modelled with a 2 s duration. To account for nuisance fluctuations in the EPI data, all first-level models included a regressor coding for global white matter drift in the MR signal (computed as mean white matter intensity after voxel-wise z-scoring). This was done rather than high-pass temporal filtering to maximize the detection power of BOLD effects that spanned across the encoding and retrieval phases of Day 1. In total, eight regressors of no interest were included in the model (one for rating (vividness), 6 for motion and one for global white matter drift). On Day 8 there were 48 regressors of interest, one for each of the 24 DelRet trials and one for each screenshot cue at the start of each retrieval phase. The DelRet regressors varied in duration and the cue was modelled with a 2 s duration. The same regressors of no interest were included in the model.

#### Within-subject RSAs

In a series of RSAs, we investigated similarities in spatial patterns of BOLD signal between encoding and retrieval phases (Enc/ImRet, Enc/DelRet), as well as between the two retrieval phases (ImRet/DelRet).

**Figure 2:**
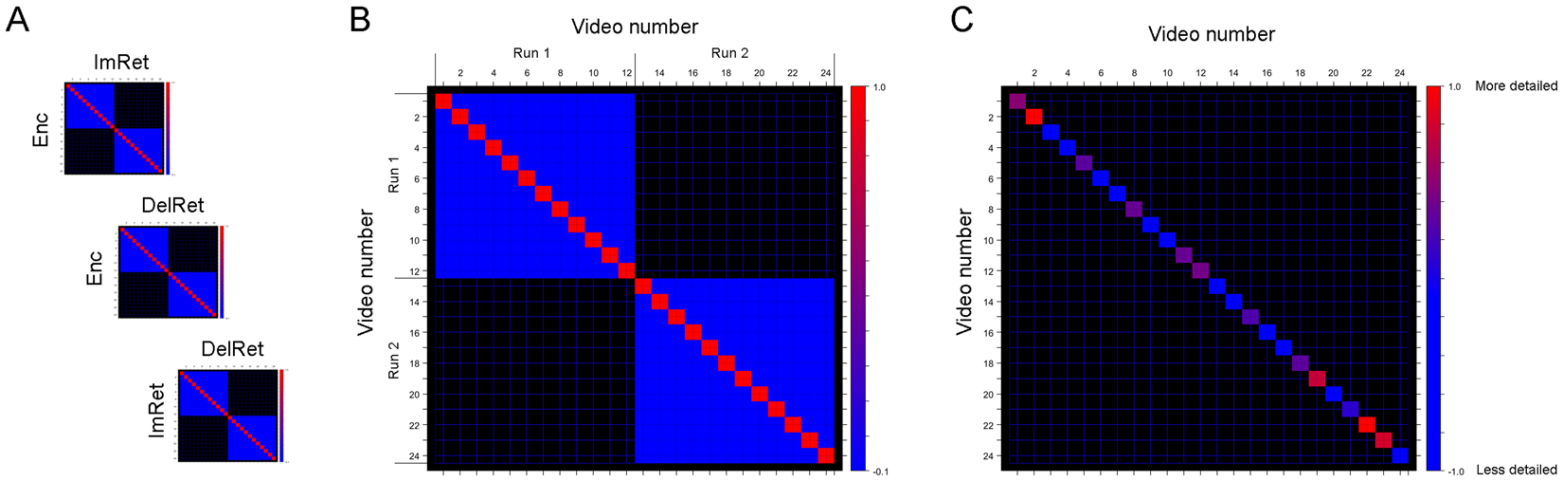
Summary of main analyses. Panel (A) Within-subject RSAs were carried out between the three phases of the experiment. Panel (B) Same- versus-different RSA. Matrix comparing pattern similarity between same- versus-different videos. Same-video correlations are shown on-diagonal (red), different–video correlations are shown off-diagonal (blue), and the weights sum to zero. Run effects within Day 1 were accounted for by not including between-run comparisons (black). Panel (C) Weighted RSA (example for a single participant). Here, only the same–video correlations along the diagonal are considered. Correlations are weighted positively and negatively by a factor of interest (e.g. memory performance) such that the value along the diagonal sums to zero. This contrast is orthogonal to the same- versus-different RSA, as it only detects regions where reinstatement is greater for videos that are weighted positively; if all videos were reinstated equally, the resulting contrast would sum to zero. Three inter-subject RSAs were also performed, corresponding to the analyses shown in Panel A. Here, matching videos from the Enc, ImRet and DelRet phases were compared between subjects rather than within subject.

In the first type of RSA, we took the correlations between activity patterns associated with the same videos and contrasted these with the correlations between different videos, i.e. a same-vs-different RSA comparison (Fig. 2A). This can be thought of as a basic reinstatement effect and might reflect general aspects of the video such as its location and the overarching theme. The second type of RSA considered only the correlations between patterns of activity for the same videos. These correlations are weighted positively and negatively by the factor of interest (memory performance or within-scanner ratings of vividness of retrieval) such that the diagonal sums to zero (Fig. 2B). This contrast identifies areas where the degree of reinstatement correlates with the richness of the retrieval.

Inputs to all searchlight analyses were *t*-statistic maps of each encoding or retrieval trial. For each type of RSA, a contrast matrix was specified that reflected the predicted differences in correlation across pairs of trials (Fig. 2). A spherical searchlight was centered at each voxel in turn and comprised all surrounding voxels within a radius of 3 voxels (=about 110 voxels on average). This searchlight returned the summed difference in Fisher-transformed correlations for each pairwise comparison of interest, and this value was assigned to the center voxel of the searchlight. The resulting images of each analysis were then normalized to MNI space (voxel size 2 × 2 × 2) and analyzed at group level in SPM8 with one-sample *t* tests against a null hypothesis of zero.

A series of within-subject RSAs are reported where we investigated the correlations in patterns of BOLD activity between Enc/ImRet, Enc/DelRet and ImRet/DelRet, both across the whole-brain and within the pre-specified ROIs. For all comparisons, we report the same-vs-different video RSA effects as well as the RSAs weighted by memory performance. We additionally report a further RSA of the retreival phases weighted by in-scanner vividness ratings. Lastly, to rule out the possibility that RSA effects during the retrieval phases were driven by processing of the cues, we report a control same-vs-different RSA, where the first 12 seconds of the retrieval trials are removed from the analysis.

#### Comparison of within-subject RSAs between the phases

To determine whether the reinstatement for ImRet/DelRet differs significantly from the reinstatement for Enc/ImRet (or Enc/DelRet), we directly compared the whole-brain maps from each RSA using paired samples *t* tests. We also compared same–video and different–video correlations from all voxels within the PMC ROI across the different phases. For the weighted analysis, we compared whole brain maps of the RSAs weighted by memory performance.

#### Inter-subject RSAs

To investigate whether there was evidence for shared neural representations of the events that are stable across time, we report inter-subject RSAs for Enc/ImRet, Enc/DelRet and ImRet/DelRet similar to those described by Chen and colleagues^12^. Following the methods of their study, we first smoothed the data using a 6mm FWHM Gaussian kernel, then normalized the data to the MNI template. Each participant’s data was then correlated using searchlight analysis with the average map of all other participants. For instance, to calculate the map for the correlation of Enc and DelRet, each participant’s smoothed map of the Enc phase for each video was correlated with the average of all other participants’ data for that video from the DelRet phase. All other methods were the same as described for the within-subjects RSAs above.

#### Comparisons of inter-subject RSAs

To investigate evidence for a systematic change in memory representations during the retrieval phases across subjects, we directly compared maps for the inter-subject RSAs between ImRet/DelRet and either Enc/ImRet or Enc/DelRet.

### Data availability statement

The datasets generated during and/or analysed during the current study are available from the corresponding author on reasonable request. Unthresholded maps for all the reported analyses are available at https://neurovault.org/collections/2814/.

## Results

### Behavioral results

We compared retrieval duration and vividness ratings for ImRet and DelRet. Retrieval duration did not change across runs, with a mean duration of 35.1 s (SD ± 8.0) during ImRet and a mean duration of 33.6 s (SD ± 8.8) during DelRet (*t*_20_ = 1.35, *p* = 0.193). Across the group of participants there was a 16% decrease in ratings of vividness over one week. Between ImRet and DelRet, vividness ratings dropped significantly from 4.2 (SD ± 0.4) to 3.5 (SD ± 0.6) (*t*_20_ = 7.7, *p* < 0.001). Based on previous studies, we expected participants to give a detailed report of videos when they recalled them a week later and indeed memory performance for each video was goo, with an average of 11.5 (SD ± 2.7) details recalled from each video. A visualization of memory performance for each participant for each video can be found in Supplementary Figure 1.

In a follow-up analysis we investigated the relationship between vividness ratings from the ImRet and DelRet trials and memory performance. At an individual level, there was a significant (*p* < 0.05) correlation between vividness ratings during ImRet and memory performance in 11 of the 21 participants. For the vividness ratings during DelRet and memory performance there was a significant correlation in 17 of the 21 participants. To analyze the relationship between vividness and memory performance across the whole group, we Fisher-transformed the Pearson correlation coefficients for each individual and tested this against 0, using a one-sample *t* test. This was significant for ImRet (*t*_20_ = 9.48, *p* < 0.001) and DelRet (*t*_20_ = 10.31, *p* < 0.001), indicating that there was a robust relationship between vividness and memory performance at the group level.

### Functional MRI results

#### Reinstatement between encoding and immediate retrieval (Enc/ImRet)

**Figure 3:**
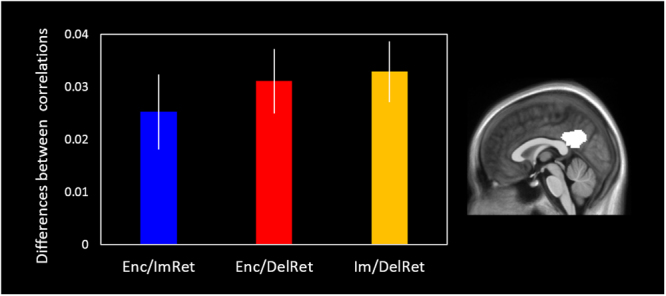
Reinstatement effects for all phases in the PMC ROI. The differences between same– versus different–video correlations are shown for the encoding and immediate and delayed retrieval phases within the PMC ROI (white). Significant effects were present in all three comparisons (the differences were greater than 0) but there were no significant differences between the effects across the three comparisons. Errors bars represent ±1 *SEM* corrected for the within subject error term.

The same- vs-different RSA between Enc and ImRet identified the precuneus bilaterally as showing a significant reinstatement effect (Supp. Figure 2 and Supp. Table 1). This replicates the finding of Bird *et al*.^11^ who showed the largest reinstatement effect between encoding and immediate retrieval in the same region. Within the independently defined PMC ROI, there was also a significant reinstatement effect (same: 0.129 (SEM ± 0.018), different: 0.104 (SEM ± 0.014), (*t*_20_ = 3.53, *p* = 0.002), see Fig. 3 below).

**Table 1:** Brain regions showing reinstatement for encoding and delayed retrieval.

Region	x	y	z	Size (voxels)	T
**RSA between encoding and delayed retrieval of the same video**					
Bilateral precuneus/right fusiform gyrus/left angular gyrus	34	−42	−18	12960	10.12
Right middle/inferior temporal gyrus	56	−20	−8	1006	6.68
**RSA between encoding and delayed retrieval of the same video weighted by memory performance**					
Right middle cingulate cortex	4	−28	36	492	4.47
Right inferior frontal gyrus	52	34	16	155	4.43

Coordinates are in MNI space. Clusters are significant at *p* < 0.05 (FWE cluster corrected; cluster-defining threshold of *p* < 0.001).

In an RSA weighted by memory performance, we identified a region in the midline of the cerebellum (Supp. Table 1). In the previous study by Bird *et al*.^11^, this analysis identified a region within the PCC. We replicated this finding: an ROI analysis within the same PCC region (277 voxels, center of mass x = −3, y = −42, z =  + 35) revealed a significant association between the strength of the Enc/ImRet correlation and memory performance (t_20_ = 2.36, *p* < 0.05).

Overall, our results for Enc/ImRet largely replicate our previous findings, showing reinstatement effects in PMC regions, both in terms of general reinstatement between encoding and retrieval as well as the degree of reinstatement correlating with subsequent memory performance within the PCC.

#### Reinstatement between encoding and delayed retrieval (Enc/DelRet)

**Figure 4:**
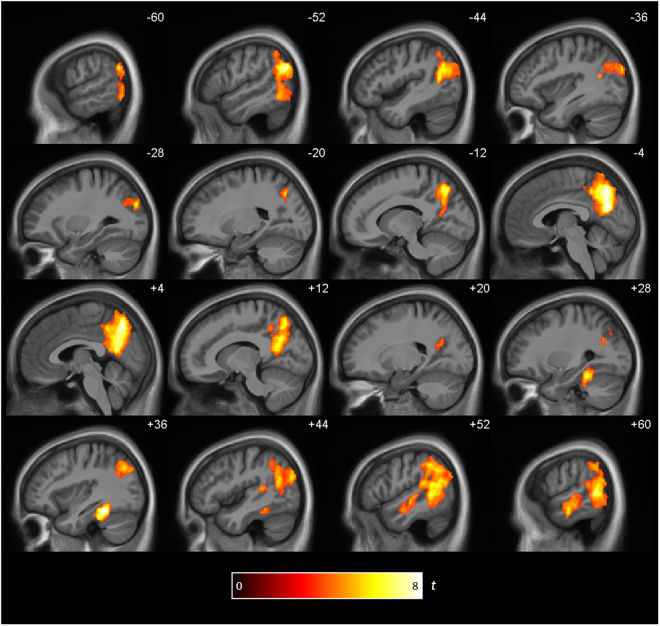
Brain regions showing reinstatement between encoding and delayed retrieval. Clusters are significant at *p* < 0.05 (FWE cluster corrected; cluster-defining threshold of *p* < 0.001.

The same- vs-different RSA for Enc/DelRet revealed a network of regions centering on precuneus and lateral temporoparietal regions (Fig. 4 and Table 1). The distribution of the reinstatement effects shown in Fig. 4 is highly similar to the reinstatement effects between encoding and immediate retrieval reported by Bird *et al*.^11^ and, at a lower threshold, to the Enc/ImRet RSA in the present study (see Supp. Figure 2). There was again a significant reinstatement effect within the PMC ROI (same: 0.131 (SEM ± 0.019), different: 0.100 (SEM ± 0.017), (*t*_20_ = 5.06, *p* < 0.001), see Fig. 3).

In a whole brain analysis two regions showed an association between the strength of the correlations between Enc/DelRet and memory performance, one in right middle cingulate cortex and one in right inferior frontal gyrus (Table 1).

Overall, these results demonstrate that even when event memories are retrieved after a week, retrieval reinstates the same patterns of activity within regions of the core retrieval network that were present during encoding.

#### Reinstatement between immediate and delayed retrieval (ImRet/DelRet)

**Figure 5:**
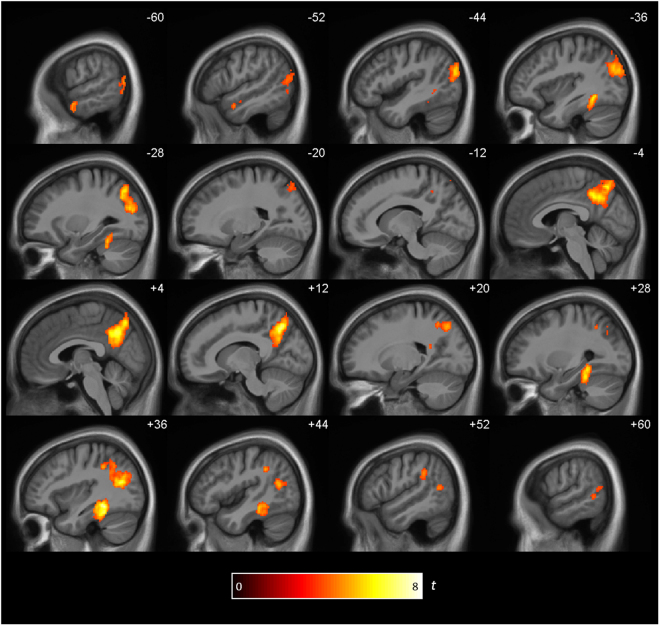
Brain regions showing reinstatement between immediate and delayed retrieval. Clusters are significant at *p* < 0.05 (FWE cluster corrected; cluster-defining threshold of *p* < 0.001).

We identified several regions where the similarities in spatial patterns of BOLD signal for ImRet/DelRet was higher for the same- versus-different videos. This reinstatement effect was found in the bilateral precuneus, left AG, bilateral MTG/occipital gyrus, bilateral fusiform gyrus and right parahippocampal gyrus (Fig. 5 and Table 2). The distribution of these reinstatement effects is very similar to those seen in the Enc/DelRet RSA (compare Fig. 5 with Fig. 4). There was once again a significant reinstatement effect within the PMC ROI (same: 0.136 (SEM ± 0.015), different: 0.103 (SEM ± 0.012), (*t*_20_ = 5.64, *p* < 0.001), see Fig. 3 below).

**Table 2:** Brain regions showing reinstatement for immediate and delayed retrieval.

Region	x	y	z	Size (voxels)	T
**RSA between immediate and delayed retrieval of the same video**					
Bilateral precuneus/left middle cingulate cortex/ right middle temporal gyrus	0	−62	36	3810	7.45
Left middle occipital gyrus/left middle temporal gyrus/left angular gyrus	−44	−80	24	1730	5.55
Right fusiform gyrus/parahippocampal gyrus	36	−42	−12	871	7.53
Left fusiform gyrus/left inferior temporal gyrus/left cerebellum	−36	−48	−12	337	6.00
Left middle temporal gyrus	−58	2	−24	167	5.80
**RSA between immediate and delayed retrieval of the same video weighted by memory performance**					
Bilateral precuneus	−8	−66	38	973	5.67
Left inferior/middle occipital gyrus/left middle temporal gyrus	−42	−72	2	689	6.38
Right inferior temporal gyrus/right middle temporal gyrus	42	−2	−30	224	5.08
Left precentral gyrus	−26	−8	52	191	4.43
Right middle temporal gyrus	44	−56	10	179	4.51
Left hippocampus/parahippocampal gyrus^a^	−22	−36	−8	35	4.95

Coordinates are in MNI space. Clusters are significant at *p* < 0.05 (FWE cluster corrected; cluster-defining threshold of *p* < 0.001). ^**a**^Within the HC/PHC ROI cluster is significant at *p* < 0.05 (FWE cluster corrected; cluster-defining threshold of *p* < 0.001).

**Figure 6:**
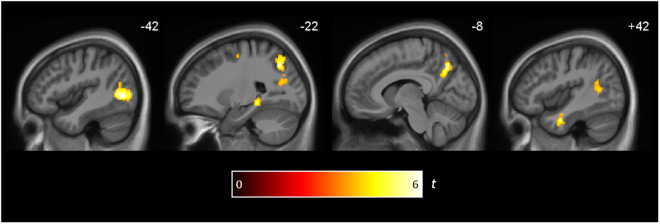
Brain regions showing reinstatement effects between immediate and delayed retrieval that correlate with memory performance. Clusters are significant at *p* < 0.05 (FWE cluster corrected across the whole brain or within a HC/PHC ROI; cluster-defining threshold of *p* < 0.001).

A number of regions showed significant effects of reinstatement for ImRet/DelRet weighted by memory performance. Most prominent were the bilateral precuneus and bilateral MTG (Fig. 6 and Table 2). Smaller regions were also identified in the right temporal pole and left precentral gyrus. Additionally, within the HC/PHC ROI there was an effect in the left posterior hippocampus/parahippocampal gyrus (*p* < 0.05 FWE cluster corrected).

A rather similar pattern of results were found when the ImRet/DelRet similarities were weighted by vividness ratings, including precuneus, bilateral MTG and right inferior temporal gyrus (see Supp. Figure 3 for a comparison). Within the PMC ROI, we found an association with ImRet/DelRet and vividness (t_20_ = 4.61, *p* < 0.001). It is unsurprising that the RSAs weighted by memory performance and vividness ratings should look similar, since both measures likely reflect the retrieval of episodic memories rich in sensory details.

It is possible that the reinstatement effects that we observed might be partly due to the presentation of a screenshot cue immediately prior to retrieval. To rule out this possibility we carried out a control same-vs-different RSA for ImRet/DelRet where we did not include the first 12 seconds of each retrieval trial. This analysis once again identified reinstatement effects in PMC, right parahippocampal gyrus/fusiform gyrus, right MTG, left inferior temporal gyrus and right AG (see Supp. Figure 4) indicating that the results were not driven by the cue.

In summary, reinstatement effects between the two retrieval phases are observed throughout the retrieval network and the pattern of effects is strikingly similar to those seen for Enc/DelRet. In several regions, reinstatement correlated with both memory performance and in-scanner ratings of vividness of retrieval, suggesting a key role of these regions in the processing of rich, sensory details. Reinstatement effects are highly unlikely to be driven by processing of the retrieval cues, since the effects are still present even if the first 12 seconds of the retrieval events are not analyzed.

#### Comparison of the patterns of reinstatement across phases

To evaluate whether reinstatement for ImRet/DelRet engaged additional regions or showed greater reinstatement compared with Enc/ImRet or Enc/DelRet, we conducted two direct comparisons between the same-vs-different RSAs. At the whole-brain level, there were no significant differences in reinstatement (see Supp. Figures 5 and 6). Within the PMC ROI, the difference between same-video and different-video correlations was similar between ImRet/DelRet and between Enc/ImRet as well as EnDelRet (Fig. 3). Importantly, there was no evidence for an interaction between correlation type (same-video and different-video) and phase (Enc/ImRet, Enc/DelRet, ImRet/DelRet) within the PMC ROI (*F*_2, 38_ = 0.53, *p* = 0.59).

Lastly, we performed whole-brain comparisons between all of the RSAs weighted by memory performance to search for regions where the association between reinstatement and subsequent memory was stronger between the ImRet/DelRet phases versus Enc/ImRet or Enc/DelRet. No regions showed this effect. Although not predicted *a priori*, the middle cingulate cortex showed a stronger effect for Enc/DelRet in contrast to ImRet/DelRet.

The purpose of these comparisons was to directly contrast the reinstatement effects for ImRet/DelRet with reinstatement between encoding and the retrieval phases (Enc/ImRet, Enc/DelRet). Identifying any region where reinstatement effects were stronger between retrieval phases compared with encoding would have been evidence for elaborated or transformed memory traces created during the immediate retrieval (retrieval practice) phase. However, we found no evidence for reinstatement between retrieval phases (ImRet/DelRet) that is over and above that seen between encoding and retrieval (Enc/ImRet, Enc/DelRet), either in terms of simple reinstatement effects (same-vs different RSA) or when reinstatement was weighted by memory performance.

#### Inter-subject RSAs

Inter-subject RSAs identified regions where the patterns of brain activity during encoding or retrieving specific videos are similar across participants, indicating a shared spatial organization of the memory representations. We performed three inter-subject RSAs (Enc/ImRet, Enc/DelRet, ImRet/DelRet), the results of which can be seen in Supplementary Figure 7. Similarly to the within-subject RSAs, these analyses identified several regions associated with the core retrieval network including the PMC, AG (as well as the supramarginal gyrus), the MTG and higher-order visual regions were associated with significant effects in all three analyses. In addition, a region including the hippocampus, parahippocampal gyrus and fusiform gyrus was identified in analyses of the correlations between Enc/ImRet (bilaterally) and Enc/DelRet (in the right hemisphere).

#### Comparisons of inter-subject RSAs

To identify whether any regions showed a consistent alteration in representations of the videos between retrieval and retrieval, we looked for brain regions in which the correlations between ImRet/DelRet were more similar than between Enc/ImRet or Enc/DelRet. The comparison of ImRet/DelRet over Enc/DelRet identified one significant effect in the middle cingulate/pre-SMA region (peak t = 6.4, x = −5, y = 7, z = 45). However, no effects were found in the PMC, AG, MTG or elsewhere in the brain. Overall the network identified with the inter-subject RSAs is remarkably similar to the core retrieval network seen across within-subject RSAs, confirming the stability of reinstatement across different individuals.

## Discussion

In this study we investigated the stability of memories for lifelike events over one week. The events were short video clips and their mnemonic representations were indexed by spatial patterns of BOLD activity measured during encoding, immediate retrieval and delayed retrieval. Consistent with previous research, inclusion of an immediate retrieval practice phase of the videos resulted in the creation of durable memories that were recalled in detail at the end of the study. Video-specific patterns of activity throughout many regions of the core retrieval network elicited during encoding were subsequently reinstated during both immediate and delayed retrieval. These patterns were not only stable within individuals but consistent across participants. No regions showed significantly greater reinstatement between the two retrieval phases compared with encoding and retrieval phases. Our findings demonstrate that detailed and vivid retrieval of an episodic memory involves reactivating representations formed during the encoding of an event, even after a week. In contrast, we did not find clear-cut evidence for an alternation in the memory representation caused by the immediate retrieval practice phase of the study.

Participants watched and silently retrieved videos on the first day of the experiment and then silently retrieved the videos again after a week, before then recalling all videos out loud. Memory performance on the final recall task was good; on average 11.5 details were recalled from each video. A previous study with a very similar procedure found that if participants were shown videos in the scanner but not asked to immediately retrieve them, they could only recall an average of 2.6 details after a week delay (^11^ Experiment 2; see also^11^ Experiment 1 and^30^ for similar findings). Furthermore, in-scanner vividness ratings taken after every retrieval event also indicated that participants were able to vividly retrieve the videos after a week. Taken together, these findings indicate that the participants performed the task as expected and we can infer that processing during the immediate retrieval results in robust memory traces that were retrieved a week later.

Memory retrieval is thought to involve the reactivation of representations that were created at the time of memory encoding^7–9^. Some of the best evidence for memory reactivation in humans comes from multivariate studies of fMRI data that have demonstrated that event-specific patterns of BOLD activity present during encoding are reinstated at retrieval^20,21,23,24^. However, these studies typically test encoding and retrieval within the same scanning session, leaving open the possibility that such effects are short-lasting and not present after the retention intervals more usually experienced outside of the laboratory. Although some studies have used longer retention intervals, in these cases the videos were extensively rehearsed^14,31^. The use of highly practiced memories meant that the memories themselves were not unique in place and time – a key feature of episodic memory^43^ (see^14^ for a discussion of this issue). In our study, we saw that reinstatement effects were robust and widespread when memories were retrieved after a week. This provides some of the strongest support to date that episodic recollection consistently reinstates memory representations created during encoding.

Our study also showed that video-specific patterns of activity are not only stable over time, but they are also consistent across participants. Within the retrieval network, video specific patterns were reinstated between participants, both when comparing encoding with retrieval and when comparing immediate with delayed retrieval. Thus, the activity patterns elicited by each video are present during encoding, immediate and delayed retrieval and share a common distribution across participants. This replicates and extends the study of Chen and colleagues^12^ who recently reported inter-subject correlations whilst participants watched and then immediately recalled an extended video.

Reinstatement effects were localized within the core retrieval network^25,26^. The effects were centered on posterior brain regions, namely bilateral precuneus, bilateral inferior lateral parietal lobe/angular gyrus and bilateral middle temporal/occipital gyrus. This network has been found in many memory retrieval studies and is also thought to guide navigation, self-referential processes, imagining the future and constructing situation models^27,44,45^. It is also affected early in the course of Alzheimer’s disease, in which episodic memory impairment is the primary symptom^46,47^. It is thought that these regions represent high-level, relatively abstract information, such as the situational content of an event, rather than low-level sensory information^12,27^.

It is possible that our reinstatement effects may reflect the activation of very broad schemas or concepts that are specific to each video. However, in the PMC, posterior hippocampus, MTG and higher-order visual regions, reinstatement between immediate and delayed retrieval correlated with memory performance outside of the scanner (Fig. 6 and Table 2). Furthermore, vividness ratings during retrieval also modulated reinstatement in a largely overlapping set of regions (see Supp. Figure 3). Our findings suggest a key role for these regions in the retrieval of rich, sensory details– the hallmark of episodic recollection. Moreover, it has been argued that active retrieval of memories, triggered by partial cues (such as the video titles used in our study), may promote the rapid consolidation of memories^10,11^. The association between reinstatement and memory performance within the core retrieval network is consistent with this proposal.

Although the effects of similarity between immediate and delayed retrieval were more widespread than between encoding and immediate retrieval (compare Fig. 5 with Supp. Figure 2), the differences between these effects were not significant (Supp. Figures 5 and 6). Within one of our primary regions of interest, the PMC, it is clear from Fig. 3 that similar reinstatement effects were observed between encoding, immediate and delayed retrieval. The inter-subject RSAs were consistent with this finding; correlations of spatial patterns of BOLD activity were similarly strong between all three phases. This implies that video-specific representations are established within regions of the core retrieval network during encoding and reinstated to a similar degree whenever the videos are retrieved.

This finding is consistent with the view that memory retrieval reactivates representations formed during encoding. However, it does not support a popular explanation for the benefits of retrieval practice: that immediate retrieval leads to a transformation or elaboration of the memory representations^33,35–37^. If this were the case, retrieval should involve the transformation of the representation of the event into one that is more generic yet reproducible^12^, or involve the incorporation of additional information^37^. In both cases, we would expect to see greater reinstatement between the retrieval phases than between encoding and retrieval – yet this was not found. Of course, it is possible that any modifications of the memory traces during immediate retrieval were undetectable in our study for a variety of reasons. What we can conclude is that the spatial and temporal grain of fMRI is well-suited to detecting the similarities in memory representations that are present both at encoding and during retrieval.

In summary, the current study shows that event-specific spatial patterns of BOLD activity established during memory encoding are reinstated during memory retrieval after as long as a week, and that these patterns are similar across individuals. This extends previous findings which have only shown reinstatement effects during the same scanning session or for highly practiced memories. In addition our study is the first to show these similarities between immediate and delayed retrieval. Although retrieval practice is known to reduce forgetting over periods of a week, we found no evidence for any systematic transformation or elaboration of the memory representations during immediate retrieval. Overall, the findings support the view that episodic recollection involves the reactivation of memory representations created during encoding even after long retention intervals.

## Electronic supplementary material

Supplementary Figures and Tables
