# Supplementary material for: Reinstatement of memory representations for lifelike events over the course of a week
Source: Sci Rep. 2017 Oct 30;7:14305. doi: 10.1038/s41598-017-13938-4 (PMC5662713; doi:10.1038/s41598-017-13938-4)
Supplement: Supplementary file 1 — Supplementary Figures and Tables [file 41598_2017_13938_MOESM1_ESM.pdf]

Reinstatement of memory representations for lifelike events over the course of a week

Christiane S.H. Oedekoven[1]
James L. Keidel[1]
Sam C. Berens[1,2]
Chris M. Bird[1]

[1]School of Psychology, University of Sussex, Brighton, UK
[2] Department of Psychology, University of York, York, UK

Supplementary Figures and Tables

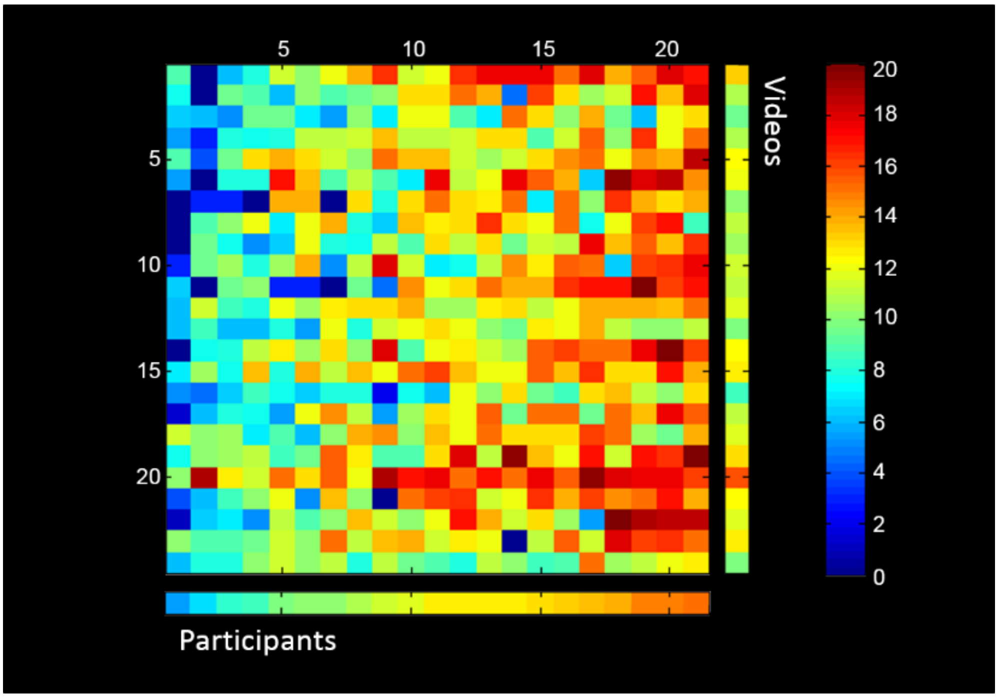

Supplementary Figure 1: Number of details recalled from each video. The 24 videos are grouped along the vertical axis. The 21 participants (horizontal) are sorted by average number of details recalled per video.

Supplementary Table 1.

Brain regions showing reinstatement for encoding and immediate retrieval

**Region**	**x**	**y**	**z**	**Size (voxels)**	**T**
**RSA between encoding and immediate retrieval of the same video**					
**Bilateral precuneus**	8	-60	38	1563	5.72
**RSA between encoding and immediate retrieval of the same video with memory performance**					
**Right cerebellum**	4	-60	-44	249	4.78

Coordinates are in MNI space. Clusters are significant at $p < 0.05$ (FWE cluster corrected; cluster-defining threshold of $p < 0.001$).

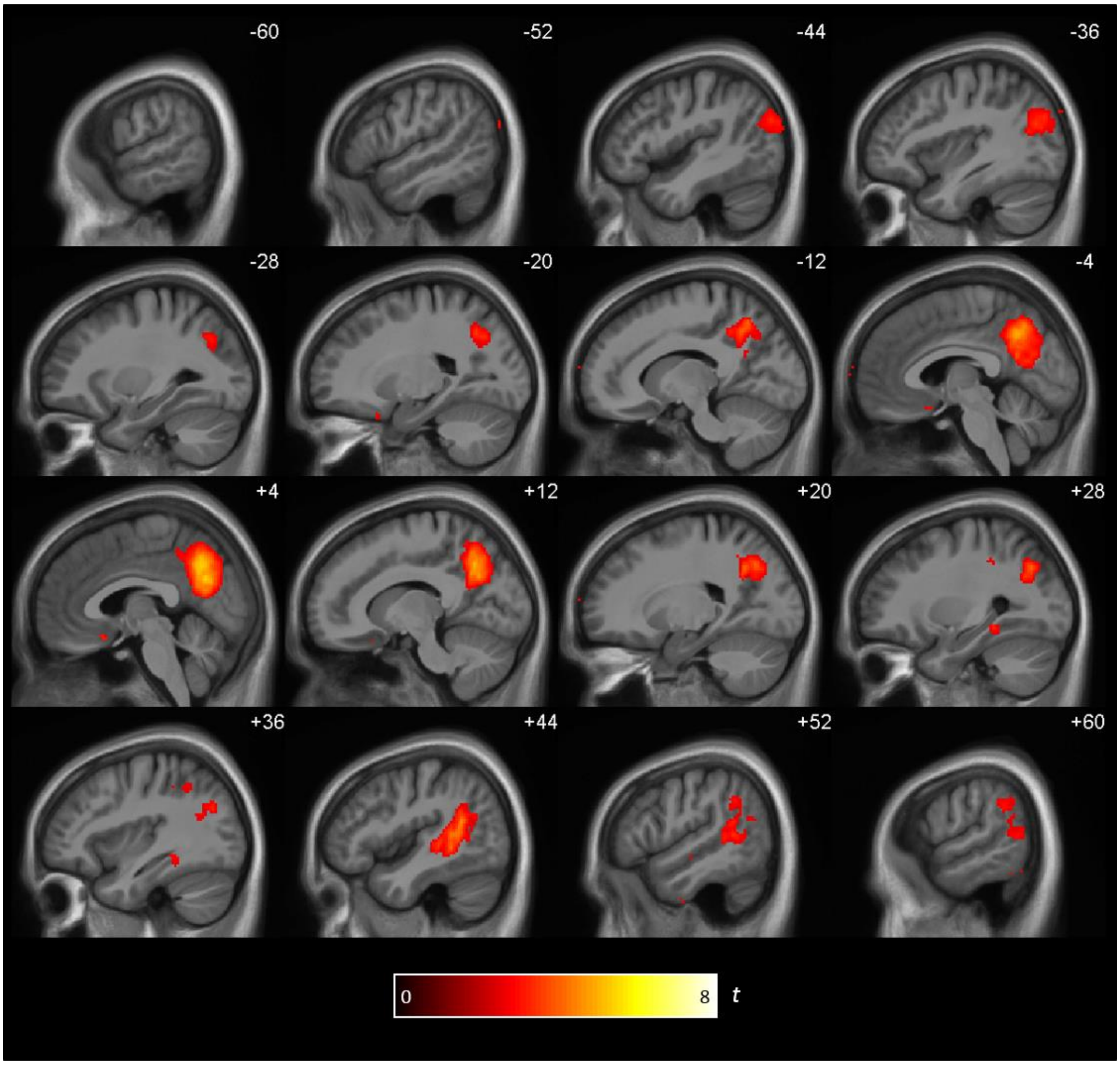

Supplementary Figure 2: Brain regions showing reinstatement between encoding and immediate retrieval. Image is thresholded at p<0.01 to illustrate the similarity in effects (at a sub-threshold level) between the same RSAs for Enc/DelRet and ImRet/DelRet displayed in the main paper. The distribution of the effects is also strikingly similar to the same analysis reported by Bird et al., (2015). Only the precuneus was significant at significant at $p < 0.05$ (FWE cluster corrected; cluster-defining threshold of $p < 0.001$).

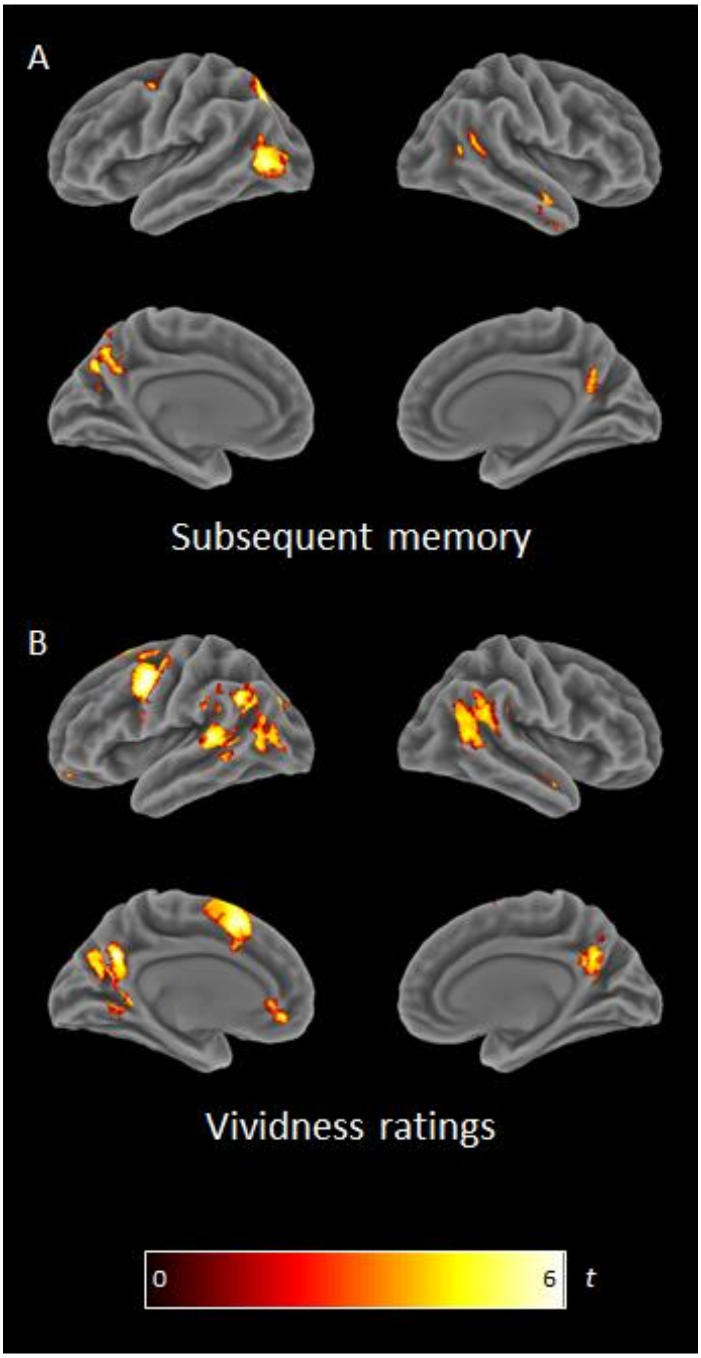

Supplementary Figure 3: Reinstatement effects between immediate and delayed retrieval weighted by A) subsequent memory performance and B) vividness ratings on Day 8. Similar effects across analyses were seen in the PMC, angular gyrus, precentral gyrus and posterior middle temporal gyrus. Clusters are significant at $p < 0.05$ (FWE cluster corrected; cluster-defining threshold of $p < 0.001$) and the results are rendered on to a standard brain in MNI space using bspmview (http://www.bobspunt.com/bspmview/).

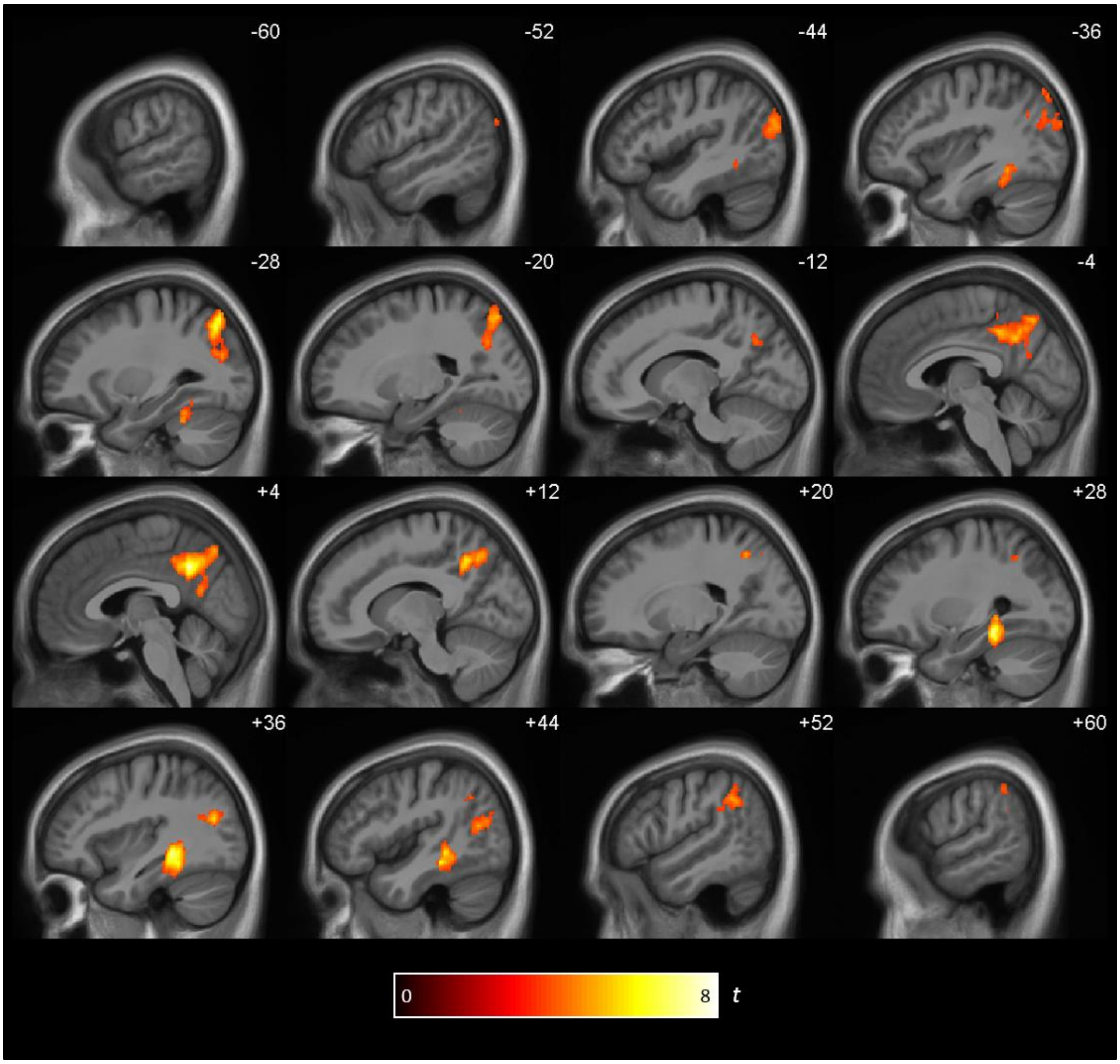

Supplementary Figure 4: Brain regions showing reinstatement effects between immediate and delayed retrieval when the first 12 seconds of each retrieval phase were removed from the model. Clusters are significant at $p < 0.05$ (FWE cluster corrected; cluster-defining threshold of $p < 0.001$).

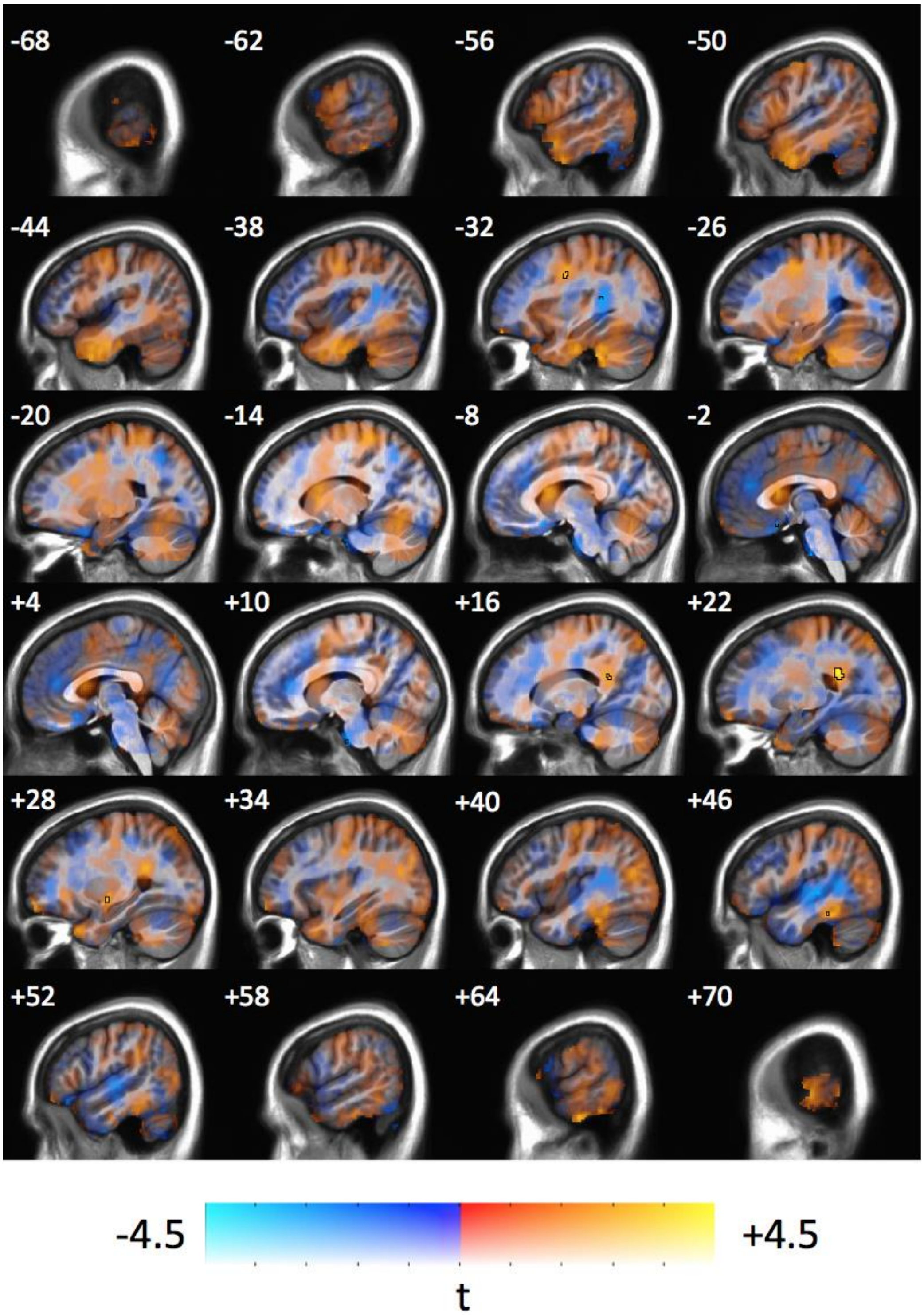

Supplementary Figure 5: Contrast of reinstatement effects: Enc/ImRet vs. ImRet/DelRet. Hue maps to the direction of the effect (cool colors = Enc/ImRet < ImRet/DelRet and Enc/ImRet > ImRet/DelRet for warm colors). Saturation of color maps to magnitude of t-statistic. Voxels with a p-value < 0.01 uncorrected are outlined in black lines. Figure follows method of Allen et al. (2012), *Neuron* 74:4.

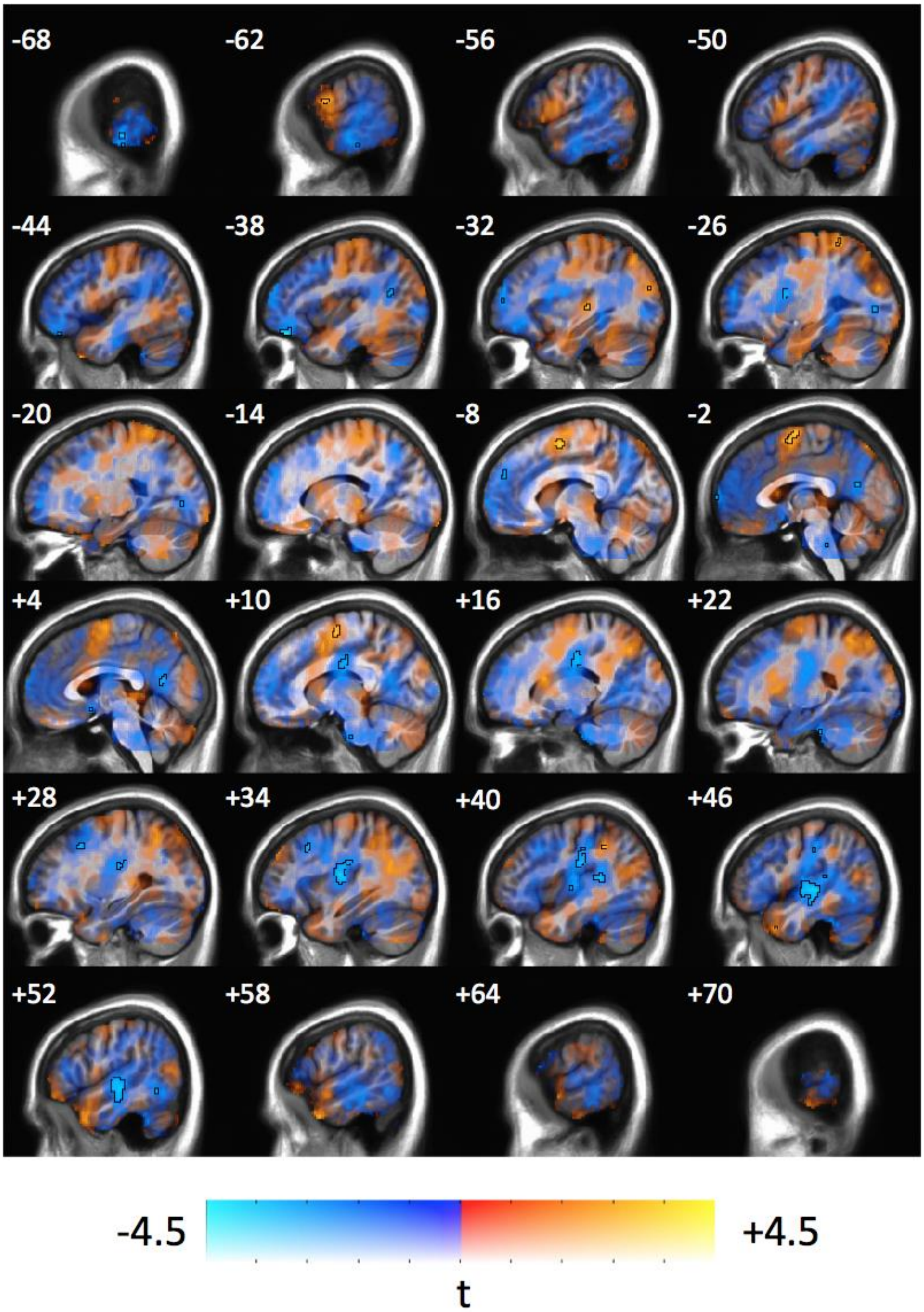

Supplementary Figure 6: Contrast of reinstatement effects: Enc/DelRet vs. ImRet/DelRet. Hue maps to the direction of the effect (cool colors = Enc/DelRet < ImRet/DelRet and Enc/DelRet > ImRet/DelRet for warm colors). Saturation of color maps to magnitude of t-statistic. Voxels with a p-value < 0.01 uncorrected are outlined in black lines. Figure follows method of Allen et al. (2012), *Neuron* 74:4.

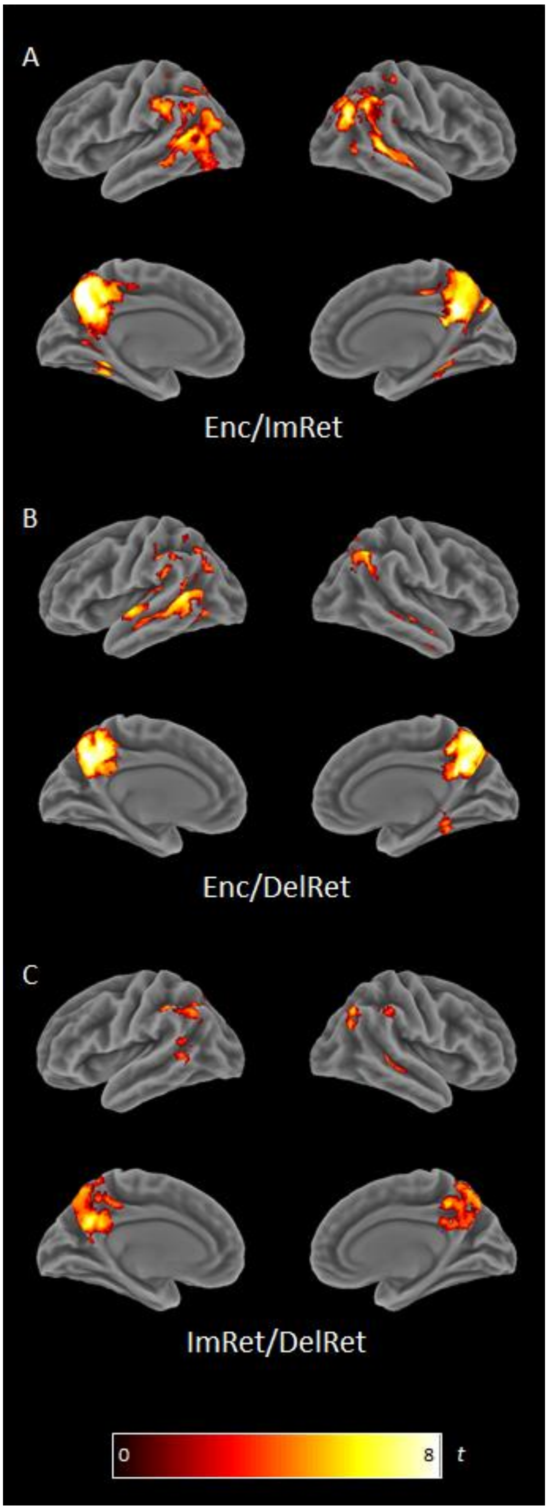

Supplementary Figure 7: Inter-subject RSAs for same- versus different videos. Each participant's map for each video was correlated with the group averaged map for the matching video. These were contrasted with the correlations between non-matching videos, as shown in Figure 2A and 2B in the main paper. Clusters are significant at p < 0.05 (FWE cluster corrected; cluster-defining threshold of $p < 0.001$).
